# Phosphorylcholine and KR12-Containing Corneal Implants in HSV-1-Infected Rabbit Corneas

**DOI:** 10.3390/pharmaceutics15061658

**Published:** 2023-06-05

**Authors:** Kamal Malhotra, Oleksiy Buznyk, Mohammad Mirazul Islam, Elle Edin, Sankar Basu, Marc Groleau, Delali Shana Dégué, Per Fagerholm, Adrien Fois, Sylvie Lesage, Jaganmohan R. Jangamreddy, Egidijus Šimoliūnas, Aneta Liszka, Hirak K. Patra, May Griffith

**Affiliations:** 1 Department of Ophthalmology, Université de Montréal, Montreal, QC H3C 3J7, Canada; 2 Maisonneuve-Rosemont Hospital Research Centre, Montreal, QC H1T 2M4, Canada; 3 Department of Clinical and Experimental Medicine, Linköping University, 58183 Linköping, Sweden; 4 Filatov Institute of Eye Diseases and Tissue Therapy of the NAMS of Ukraine, 65061 Odessa, Ukraine; 5 Institute of Biomedical Engineering, Université de Montréal, Montreal, QC H3T 1J4, Canada; 6 Department of Microbiology, Asutosh College, Affiliated with University of Calcutta, Kolkata 700026, India; 7 Département de Microbiologie, Infectiologie et Immunologie, Université de Montréal, Montreal, QC H3T 1J4, Canada; 8 Department of Biological Models, Institute of Biochemistry, Life Sciences Center, Vilnius University, 01513 Vilnius, Lithuania; 9 Department of Surgical Biotechnology, UCL Division of Surgery and Interventional Science, University College London, London WC1E 6BT, UK

**Keywords:** HSV-1 infection, corneal implant, RHCIII-MPC, KR12, nanoparticles, rabbits, regeneration

## Abstract

Severe HSV-1 infection can cause blindness due to tissue damage from severe inflammation. Due to the high risk of graft failure in HSV-1-infected individuals, cornea transplantation to restore vision is often contraindicated. We tested the capacity for cell-free biosynthetic implants made from recombinant human collagen type III and 2-methacryloyloxyethyl phosphorylcholine (RHCIII-MPC) to suppress inflammation and promote tissue regeneration in the damaged corneas. To block viral reactivation, we incorporated silica dioxide nanoparticles releasing KR12, the small bioactive core fragment of LL37, an innate cationic host defense peptide produced by corneal cells. KR12 is more reactive and smaller than LL37, so more KR12 molecules can be incorporated into nanoparticles for delivery. Unlike LL37, which was cytotoxic, KR12 was cell-friendly and showed little cytotoxicity at doses that blocked HSV-1 activity in vitro, instead enabling rapid wound closure in cultures of human epithelial cells. Composite implants released KR12 for up to 3 weeks in vitro. The implant was also tested in vivo on HSV-1-infected rabbit corneas where it was grafted by anterior lamellar keratoplasty. Adding KR12 to RHCIII-MPC did not reduce HSV-1 viral loads or the inflammation resulting in neovascularization. Nevertheless, the composite implants reduced viral spread sufficiently to allow stable corneal epithelium, stroma, and nerve regeneration over a 6-month observation period.

## 1. Introduction

Herpes Simplex Virus serotype 1 (HSV-1) infection is the leading infectious cause of unilateral corneal blindness in developed nations and the third most common indication for patients requiring corneal transplantation worldwide [1,2,3,4,5,6]. A million new and 9,000,000 recurrent cases are reported each year globally [7]. Severe infections are accompanied by inflammation or keratitis (Herpes Simplex Keratitis or HSK), which can cause scarring, neovascularization, or even perforation, resulting in blindness. Adult infections are usually unilateral, but bilateral infections occur in children and in the setting of atopy [7]. Primary HSV infections generally only affect the epithelium and may be undetected, recovering with minimal effects on vision [8,9]. However, the virus remains in the body after establishing latency in the trigeminal ganglia. Reactivated HSV-1 causes recurrent disease leading to inflammation and HSK and can involve all layers of the cornea [9]. The antiviral drug Acyclovir (ACV) and its derivatives are used to combat the viral infection while steroids are used to suppress inflammation. As the uptake of ACV through the corneal epithelium is extremely poor, treatment is given systemically, necessitating high doses. Prophylactic use is ineffective, e.g., 23.1% of patients on a 12-month treatment regime of high daily doses of 800 mg ACV or 500 mg of its prodrug, valacyclovir (V-ACV), reported recurrent disease [10,11]. Moreover, the long-term use side effects include nausea and headache, and ACV resistance is documented [12]. Ganciclovir, a potent ACV derivative, is used as a standard-of-care ophthalmic gel for treating acute herpes keratitis symptoms in the US and other countries but is not a cure [13].

Cationic host defense peptides (CHDPs) like defensins and cathelicidins are part of the innate immune system. They have antibacterial and antiviral activity as well as immunomodulatory activity and are being examined as potential anti-infective drugs [14]. There is only one human cathelicidin, the human cationic antimicrobial peptide of 18 kDa (hCAP18). The hCAP18 peptide is produced by various epithelial cells, including corneal cells, and is cleaved by serine proteases to release LL37, a 37-amino acid segment with potent antiviral activity against HSV-1 [15]. Although the exact mechanism of the antiviral action is poorly understood, disruption of the viral envelope and prevention of viral entry are probably involved. In Lee et al. [16], we confirmed that LL37 blocked HSV-1 entry into corneal cells in vitro but could not wholly prevent viral replication or spread in pre-infected cells. Here, we decided to use smaller LL37 fragments containing the sequences critical to its anti-infective properties, selecting KR12 (KRIVQRIKDFLR), the smallest LL37 fragment with anti-infective properties against bacteria [17]. Being smaller, we could load higher concentrations of peptide for delivery. We tested the biocompatibility and efficacy of KR12 against the McKrae strain of HSV-1 in vitro. This strain was isolated from a patient with corneal HSK and is the strain commonly used to establish rabbit models of corneal HSK [18].

We previously showed that cell-free corneal hydrogel implants made from recombinant human collagen type III and 2-methacryloyloxyethyl phosphorylcholine (RHCIII-MPC) promoted in situ tissue regeneration when grafted into the corneas of patients at high risk for rejecting conventional human donor allografts [19]. High-risk factors include neovascularization, neolymphangiogenesis, and heightened inflammatory responses due to previous inflammatory, allergic, or infectious causes such as HSK [20]. Being cell-free, the implants do not trigger sensitization of allogeneic cells [21], and polymeric MPC has been reported to have inflammation-suppressing properties [22,23]. However, while collagen-MPC hydrogels performed better than mismatched corneal allografts, they were nevertheless rejected at 22 days post-operation in HSK mice [16].

We therefore tested the hypothesis that composite RHCIII-MPC hydrogels with inflammation-suppressing MPC, incorporating a CHDP like KR12 that can block HSV-1 activity, will suppress viral activity sufficiently to allow stable regeneration.

We encapsulated KR12 within transparent silica dioxide nanoparticles (SiNPs), incorporated these into RHCIII-MPC implants, and tested the efficacy of the composite implants in HSV-1-infected rabbit corneas. Mesoporous SiNPs are used for controlled, targeted drug delivery due to their low toxicity [24] and are “Generally Recognized as Safe” by the United States Food and Drug Administration [25]. SiNPs are more stable to external responses such as degradation and mechanical stress than liposomes, niosomes, or polymeric nanoparticles [24,26,27]. The potential safety and optical clarity of SiNPs were essential considerations for their use in the transparent cornea. Progress in the regeneration of corneal tissue and nerves, and virus shedding was monitored over six months.

## 2. Materials and Methods

### 2.1. In Silico Comparison of KR12 and LL37

In silico modeling was performed to test the assumption that KR12 is more reactive than its parent LL37 molecule for interacting with HSV-1 in the mammalian body, an aqueous milieu. The accessibility or Residue Given Burial (*rGb*) score of a peptide estimates how stable or reactive it will be in an aqueous environment [28,29,30,31]. *rGb* was used to compare the hydrophobic burial profiles of KR12 and LL37 with respect to their general trends (or corresponding native distributions) as enumerated from standard globular protein databases. The function measures the distribution of amino acid residues in a folded protein (or peptide) chain (or associated chains) as a function of solvent exposure and computes its compatibility fitness (or the extent of stability) in an aqueous environment. It is an integral part of the complementarity plot [28]. The score is analytically designed and based on the normalized conditional probability (or propensity) estimates of each amino acid (e.g., valine (Val), asparagine (Asn), histidine (His), etc.) given their burial (and hence the name: *rGb*: *residue Given burial*). The score is calculated as follows:

$$rGb=\frac{1}{N_{res}}\sum_{i=1}^{N_{res}}{log}_{10}\left({Pr}_{i}\right)$$

where *N_res_* is the sequence length of the input polypeptide chain and *Pr_i_* is the propensity of a particular amino acid (Val, Asn, His, etc.) to acquire a particular degree of exposure to aqueous solvent. A value of *rGb* > 0.011 [28] (the higher the better) renders the input atomic model affirmative with regard to the ‘native-like’ distribution of amino acids in terms of their hydrophobic burial. However, a value of less than 0.011 means that the hydrophobic residues are exposed to the solvent, causing the molecule to remain in an unfavorable/frustrated disordered (high-entropy) state. A negative value is indicative of severe instability and hence, the reaction-prone nature of a protein or peptide.

Before calculating the *rGb* score, the solvent-accessible surface areas (ASA) were calculated using NACCESS [32] following the standard Lee and Richards protocol [33]. The burial (bur) of ASA for each amino acid residue pertaining to the peptide(s) was computed following standard procedures [34].

The membrane propensity (MPr) index [35] describes the membrane (or lipidic) preference of a given protein or peptide. The index was primarily designed to identify stability strength or weaknesses in the trans- and extra-membrane regions of membrane proteins. However, its application is not restricted to membrane proteins, but rather, it can be used to indirectly interpret the aqueous preference of globular protein units. The higher the MPr score of a protein, the higher its preference (stability) for lipid environments, while the lower the MPr score, the higher its preference (stability) in aqueous environments [31,35]. The peptide MPr scores were obtained directly from the BRANEart web-interface (http://babylone.3bio.ulb.ac.be/BRANEart/index.php; accessed on 15 March 2022) along with realistic visual representations of the structures colored according to the stability index, MPr.

### 2.2. Effect of KR12 on HSV-1 and Corneal Cells

#### 2.2.1. In Vitro Cell Viability Studies

To determine the KR12 biocompatibility/cytotoxicity, a line of immortalized human corneal epithelial cells (HCECs) that retains key characteristics of primary cells [36] was used. HCECs were seeded at a density of 2 × 10^4^ cells/well in a 48-well tissue culture plate supplemented with Keratinocyte Serum Free Media containing bovine pituitary extract and epidermal growth factor (EGF) supplements (KSFM; Life Technologies, Invitrogen, Paisley, UK) and allowed to attach. After an initial KR12 dilution range to obtain the inhibition concentration where 50% of cells are killed (IC50), HCECs were exposed to a smaller range of concentrations of KR12 peptide (5, 10, 15, 25, and 50 µM) diluted in cell culture media for 24 and 48 h. LL37 peptide was used as a benchmark while KSFM alone served as a negative control. Alamar Blue™ cell viability reagent (Invitrogen, Thermo Fisher Scientific, Eugene, OR, USA) was added to the culture for 3 h before media collection to allow for appropriate incubation times. Absorbance was read at 570 nm on a microplate reader (Tecan Spark, Tecan Austria GmbH Untersbergstr., Grödig, Austria) using 600 nm as a reference wavelength according to the manufacturer’s direction.

#### 2.2.2. Wound Healing Assay

A wound healing assay was performed to ensure that cells were not only viable in the presence of KR12 but were also able to multiply and fill a potential wound gape in vivo. Green fluorescence protein (GFP) transfected HCECs [37] were used to facilitate cell imaging. The GFP-HCECs were cultured in 12 well plates. At confluency, the cell monolayers were scratched in a straight line across each well with a 200 µL pipet tip. Cells were incubated with KSFM without EGF and supplemented instead with either KR12 or LL37 peptides at concentrations of 5, 10, and 15 µM in 0.01 M phosphate buffered saline (PBS). The width of the scratch was examined at three time points (0, 24, and 48 h) by taking images with an LSM700 confocal microscope (Carl Zeiss AB, Stockholm, Sweden). Positive controls for wound healing comprised cells that were grown in the presence of EGF (5, 10, 20 ng/mL), which is known to support corneal epithelial wound healing. Negative controls were supplemented with PBS.

#### 2.2.3. In Vitro Efficacy Studies of KR12 Peptide

To determine the in vitro efficacy of KR12 peptide in blocking HSV-1 entry into cells, HCECs were seeded in 48-well plates supplemented with DMEM containing 10% serum until 90% confluency was reached. They were infected with HSV-1 (McKrae strain) in serum-free DMEM at a multiplicity of infection (MOI) of 1 in the presence of varying concentrations of KR12 peptide. Preliminary experiments were performed to obtain the effective concentration at which there is a 50% reduction (EC50) in plaque-forming units (PFUs). From there, a range of 0, 5, 10, 25, and 50 µM KR12 peptide was added into the HCEC cultures simultaneously with the HSV-1 inoculation. After one hour to allow adsorption of the virus onto cells [16], the unattached viruses were washed away. The cells were subsequently cultured in fresh media containing similar concentrations of KR12. At 24 and 48 h after infection, the infected cell culture media was collected to quantify PFUs of viruses that remained after KR12 treatment using the liquid overlay method [38].

The plaque assay was performed with Vero cells seeded in 12-well plates and supplemented with DMEM containing 10% FBS until 90% confluency. The Vero cells were infected with the 24 h and 48 h post-infection media from HCECs. After 1 h of incubation, the infected media was replaced with Avicel (Millipore—Sigma, Oakville, ON, Canada): DMEM media (1:1) and incubated for 3 days to obtain clear plaques. Cells were fixed with 10% formaldehyde, stained with crystal violet, and counted for PFUs [38]. The infection ratio (%) was calculated as (plaque numbers in treated samples/plaque numbers in untreated samples) × 100%.

### 2.3. Silica Nanoparticles Releasing KR12 and Composite RHCIII-MPC Implants with KR12 Activity

KR12 was incorporated into silica nanoparticles (SiNPs) for sustained antiviral drug release. SiNPs were prepared using a modification of our previously described method [16]. Briefly, 6 mL of cyclohexane (Acros Organics, Thermo Scientific, Waltham, MA, USA) was combined with 2 mL Triton X-100 (Sigma-Aldrich Chemie GmbH, Schnelldorf, Germany), followed by the addition of 1 mL H_2_O containing 7.68 mg of KR12 (Vildoma, Vilnius, Lithuania). Under magnetic stirring, 0.75 mL tetraethylorthosilicate (Sigma-Aldrich Chemie GmbH, Schnelldorf, Germany) was added dropwise, followed by slow neutralization of the mixture using concentrated ammonia hydroxide (Merck KgaA, Darmstadt, Germany) to reach a target pH of 5.0–6.0. The mixture was stirred for two days at 50 °C. The resulting SiNPs containing KR12 were washed twice with 50% ethanol and vacuum dried. The particle size and morphology were determined by transmission electron microscopy (TEM) using a JEOL1230 Transmission Electron Microscope.

RHCIII-MPC implants were fabricated under aseptic conditions in a laminar flow hood as previously described [19]. Briefly, implants were fabricated by mixing a 16% (wt/wt) aqueous solution of RHCIII (FibroGen Inc., San Francisco, CA, USA) with 2-methacryloyloxyethyl phosphorylcholine (MPC, Paramount Fine Chemicals Co. Ltd., Dalian, China) and poly (ethylene glycol) diacrylate (PEGDA, Mn 575, Sigma-Aldrich) in a morpholinoethane sulfonic acid monohydrate (MES, Sigma-Aldrich, St. Louis, MO, USA) buffer. The ratio of RHCIII:MPC was 2:1 (wt/wt) and PEGDA:MPC was 1:3 (wt/wt). To the homogenous, bubble-free mixture, 300 µL MES containing 19 mg of SiO_2_ encapsulating KR12 peptide was combined. After ensuring thorough mixing, polymerization initiators, ammonium persulphate (APS; Sigma-Aldrich) and N,N,N,N-tetramethylethylenediamine (TEMED, Sigma-Aldrich), at ratios of APS:MPC = 0.03:1 (wt/wt), APS:TEMED (wt/wt) = 1:0.77), crosslinker, N-(3-dimethylaminopropyl)-N′-ethylcarbodiimide (EDC; Sigma-Aldrich) and its co-reactant, N-hydroxysuccinimide (NHS; Sigma-Aldrich) were mixed in. The resulting solution was dispensed between flat glass plates with 500 µm spacers (for in vitro studies) and cured. For in vivo implantation into rabbits, the composite mixtures were dispensed into curved cornea-shaped molds (12 mm diameter, 350 µm thick). After demolding, the hydrogels were washed thoroughly with phosphate-buffered saline (PBS) containing gentamicin 250 µg/mL and amphotericin 12.5 µg/mL and placed into empty sterile vials that were sealed to maintain sterility and stored at −80 °C to prevent premature peptide release from the implants. Implant sterility was confirmed by a 3rd party laboratory (APL, Stockholm, Sweden).

#### 2.3.1. KR12 Release Study and Encapsulation Efficacy

To characterize KR12 release from SiNPs, 20 mg of empty NPs and NPs containing KR12 were incubated in 1.5 mL PBS solution in 12-well plates at 37 °C under continuous mechanical shaking for 30 days. N = 3 independent samples. Fluorescent Alexa 488-tagged KR12 (Vildoma, Vilnius, Lithuania) was used for the peptide release assay. On Days 1, 3, 7, 10, 14, 21, and 30, PBS was collected and replaced by fresh PBS. All the collected PBS at different time points as well as supernatants collected after NPs washing were read at 485 nm on Victor X4 multimode plate reader (PerkinElmer, Waltham, MA, USA).

To check KR12 release from the implants, implants containing SiNP encapsulated fluorescently-tagged KR12 were placed in 10 mL PBS at 37 °C under continuous mechanical shaking for 21 days and assayed for amounts of peptide released. On Days 1, 3, 7, 10, 14, and 21, the PBS was collected and replaced by fresh PBS. All PBS time points were analyzed using a Victor X4 multimode plate reader as described above.

KR12 quantification was performed based on a calibration curve constructed by analyzing standard samples of KR12 with the identical method. To determine the efficacy of KR12 loading, the following equations were used:

Encapsulation efficacy (%) = ((total peptide weight − free peptide weight)/total peptide weight) × 100%

Loading efficacy (%) = ((total peptide weight − free peptide weight)/particles weight) × 100%

Note: total peptide weight refers to weight of KR12 used for encapsulation; free peptide weight refers to weight of KR12 in supernatants collected during particle washing.

#### 2.3.2. Biocompatibility of KR12-Releasing RHCIII-MPC Hydrogels In Vitro

To assess whether RHCIII-MPC hydrogels incorporating KR12-releasing SiNPs are cytotoxic or not, a 3-(4,5-dimethylthiazol-2-yl)-2,5-diphenyl-2H-tetrazolium bromide (MTT) colorimetric assay for cell metabolic activity as an indicator of cell viability and proliferation was performed. Green fluorescence protein-labeled human corneal epithelial cells (GFP-HCECs) were used [37]. In brief, 1 × 10^4^ GFP-HCECs were seeded on the top of 6 mm diameter implants in 96-well plate cultured for 48 h using a 37 °C, CO_2_ incubator. An MTT assay was performed: 10 μL of MTT reagent (Merck KGaA, Darmstadt, Germany) at a concentration of 10 mg/mL was added to each well and incubated for 4 h at 37 °C in CO_2_ incubator. The plate was centrifuged, the supernatant was removed, and 150 μL of 50% ethanol solution in dimethyl sulfoxide was added to each well. The plate was read at 570/630 nm using a VersaMax ELISA microplate reader (Molecular Devices, Sunnyvale, CA, USA). Triplicate measurements were made.

We also examined the effects of KR12-releasing hydrogels on bone marrow dendritic cells (BMDCs). With ethical permission from the Animal Care and Use Committee of Maisonneuve-Rosemont Hospital, the tibia and femur of six male C57BL/6J mice (6 to 12 weeks old) were used to obtain BMDCs [39]. One million cells per mL were seeded into 6-well suspension culture plates with RPMI 1640 containing 10% (*v*/*v*) fetal bovine serum (FBS) (Wisent), penicillin-streptomycin-glutamine (0.5 mg/mL), 10 mM HEPES, 1 mM sodium pyruvate, 55 μM of β-mercaptoethanol, and granulocyte-macrophage colony-stimulating factor (2.5 ng/mL; GM-CSF) (all from Gibco, Thermo Fisher Scientific, Waltham, MA, USA). At 2 and 3 days after seeding the cells, half the media was exchanged with fresh media containing GM-CSF (5.0 ng/mL). Cells were collected at day 6 and a Histodenz density gradient (Sigma-Aldrich, St. Louis, MO, USA) was used to isolate enlarged cells. These cells were plated at a density of a million cells per ml in 24-well suspension culture plates. BMDCs were incubated for 24 h with either a 6 mm diameter, 500 μm thick hydrogel disk, or 1.27 mg of SiNP encapsulated KR12. Lipopolysaccharides (LPS) from *Escherichia coli* O26:B6 (L2654-1MG, Sigma-Aldrich, St. Louis, MO, USA) were used as a positive control to activate BMDCs. BMDCs were stained using directly conjugated antibodies against CD11c, CD40, CD80, and CD86 (Table 1). A Zombie Aqua Fixable Viability Kit (BioLegend, San Diego, CA) was used to identify the live cells while CD11c staining was used to identify dendritic cells. A BD LSR II flow cytometer was used to acquire data that was analyzed using FlowJo software (Becton, Dickinson and Company, Franklin Lakes, NJ, USA). BMDCs were identified based on their expression of Zombie Aqua and CD11c. Mean fluorescence for CD40, CD80, and CD86 for each condition was compared to untreated cells.

### 2.4. Rabbit HSV-1 Infections and Surgery

**Figure 1:**
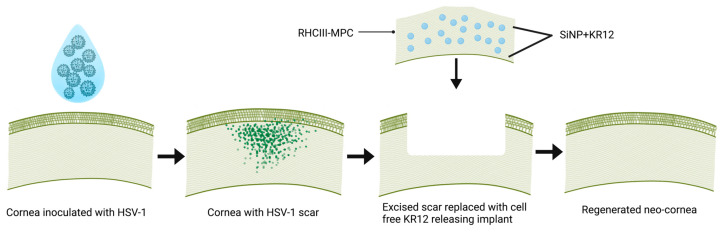
Schematic summarizing the in vivo rabbit study design. Each rabbit cornea was infected with HSV-1 and allowed to heal and scar. Next, scar revision was performed by excising the damaged corneal epithelium and stroma by anterior lamellar keratoplasty and implanting a cell-free RHCIII-MPC implant containing SiNPs releasing KR12. The rabbits were followed post-surgically to monitor neo-corneal regeneration.

The rabbit study was performed at the Karolinska Institutet Astrid Fagræus Laboratory facility for animal research on infectious diseases in humans, Stockholm, Sweden. Figure 1 is a schematic that summarizes the rabbit study. In compliance with the Swedish Animal Welfare Ordinance and the Animal Welfare Act, and with ethical permission from the local ethical committee (Stockholms Djurförsöksetiska Nämnd), 12 New Zealand rabbits weighing from 3.1 to 3.7 kg were anesthetized with ketamine 100 mg/mL (0.35 mL/kg) and xylazine 20 mg/mL (or medetomidine 1 mg/mL) (0.25 mL/kg). The McKrae strain of the virus, a gift from D.J. Carr, Univ. of Oklahoma Health Sciences Centre, Oklahoma City, OK, USA, was used to establish the HSV-1 infection. The right cornea of each animal was marked centrally using a 5 mm diameter trephine. Scratches were made in the epithelium within the circular mark using a 30 G needle, after which one 10 μL aliquot of virus (1 × 10^5^ ± 500 PFU) was applied. Of the 12 animals that were infected, eight animals were euthanized after developing symptoms of encephalitis.

The infected and scarred central corneas of the remaining four rabbits were operated on by anterior lamellar keratoplasty 3 months after primary infection. The anterior part of the cornea (approximately 80% thickness) was excised by trephining with a 6.5 mm blade set to a depth of 300 μm, followed by dissection using a surgical blade. A 6.75 mm diameter and 350 μm thick composite implant was placed into each surgical wound bed and secured with 3 overlying sutures using 10/0 nylon. Immediately after implantation, two of the rabbits received an additional dose of 5000 PFU HSV-1 each to mimic peri-surgical virus re-activation in their respective corneas.

All rabbits were given a combined hydrocortisone (10 mg/g of gel) and oxytetracycline (5 mg/g of gel) ointment (Pfizer AB, Sollentuna, Sweden) after the surgery. Post-surgical management included combined topical eyedrops of tobramycin 3 mg/mL and dexamethasone 1 mg/mL, applied 2 times daily during the first week post-operation. Sutures were removed at 1 month post-operation. The animals were followed-up for 6 months. Clinical evaluation of both eyes included slit-lamp biomicroscopy to evaluate the corneas and the implants for optical clarity/haze and any inflammation (as indicated by corneal neovascularisation, excessive conjunctival redness, swelling, and changes in aqueous humor transparency compared to the contralateral unoperated and uninfected control eyes) using a modified MacDonald–Shadduck scoring system [40]. Other tests included intraocular pressure (IOP) measurements (TonoVet, Icare Finland Oy, Vantaa, Finland), sodium fluorescein staining to assess epithelial integrity, ultrasound pachymetry (Tomey SP 3000, Tomey, Inc., Nagoya, Japan) to check corneal thickness and Schirmer’s test to assess tear production (Haag Streit UK Ltd., Harlow Essex, UK). In vivo confocal microscopy (IVCM; Heidelberg Engineering GmbH, Dossenheim, Germany) was performed on both corneas of each animal before infection (to obtain a baseline) and at three and six months after surgery to monitor the cell-implant interaction.

#### 2.4.1. Histopathology and Immunohistochemistry

At six months post-operation, the rabbits were euthanized. The corneas were excised with the adjacent limbal area and 2 mm of scleral rim and fixed in 4% paraformaldehyde in 0.1 M PBS, at pH 7.2–7.4. The samples were processed for conventional haematoxylin-eosin (H&E) staining for histopathological examination and immunohistochemistry. Immunohistochemical staining of cryosectioned treated and control corneas was performed with primary antibodies purchased from AbCam (Cambridge, UK). Details of the antibodies are shown in Table 2.

Briefly, the cornea samples collected onto glass slides were doubly fixed with 4% PFA (10 min, +4 °C) and cold methanol (10 min, −20 °C), air dried, immersed in 0.01 M PBS, permeabilized with 0.3% Triton X-100 in 0.01 M PBS for 10 min at room temperature (RT), and then blocked with 5% goat serum and 0.1% saponin in 0.01M PBS (blocking solution) for 60 min at RT. Next, samples were incubated overnight at 4 °C with the primary antibodies diluted with the blocking solution. All slides were washed in PBS containing 1% Tween 20 (PBS-T) and incubated with the secondary antibodies (goat anti-mouse Alexa 488, goat anti-rabbit Alexa 594, or goat anti-mouse Alexa 594; Jackson ImmunoResearch Laboratories Inc., West Grove, PA, USA) diluted 1:1000 with the blocking solution for 60 min at RT. After washing in PBS-T, the slides were dehydrated and mounted with Vectashield mounting medium with DAPI (Vector Laboratories, Inc., Burlingame, CA, USA). Images were captured on a Zeiss LSM800 Zeiss confocal laser-scanning microscope (Carl Zeiss, Göttingen, Germany).

#### 2.4.2. Real-Time Quantitative PCR to Check HSV-1 Shedding in Rabbit Tears

The relative change in HSV-1 shedding over time was analyzed by real-time quantitative polymerase chain reaction (PCR) following Bustin et al. [41]. Tears from both eyes of all rabbits were collected before HSV-1 inoculation, and then weekly until the end of the study. Schirmer tear test strips (Haag-Streit UK Ltd., Harlow, UK) were placed into lower conjunctival fornix of each eye and left long enough to wet the strip at least 5 mm. Strips were stored in sterile Eppendorf tubes at −80 °C before DNA extraction.

For DNA extraction, the tear-soaked strips were cut into 2-mm widths, immersed in 250 µL 0.01 M phosphate-buffered saline (PBS) solution, heated at 72 °C for 15 min, and incubated at RT for 4 h. Finally, solution was collected after centrifugation at 10,000 rpm for 1 min at RT and DNA was extracted by QIAmp DNA Mini Kit (QIAGEN GmbH, Hilden, Germany) according to manufacturer’s instructions. DNA concentration in elution solution was checked using spectrophotometer NanoDrop 1000 (Thermo Fisher Scientific, Wilmington, DE, USA) according to manufacturer instructions. For positive controls, 30–50 µL HSV-1 strain F stock solution containing 2 × 10^8^ PFU/mL was used for DNA extraction; for negative controls, new Schirmer strips were handled in similar manner to the tested ones.

For DNA amplification, primers for HSV-1 immediate-early regulatory protein ICP27 that are needed for infection and a fluorescent probe (IDT, Leuven, Belgium) were used. The forward and reverse primer sequences were 5′-CCTTTCTCCAGTGCTACCTG-3′ and 5′-GCCAGAATGACAAACACGAAG-3′, respectively. The fluorescent probe sequence was 5′-6FAM/TG TCC TTA A/Zen/T GTC CGC CAG ACG C/3IABkFQ-3′. All reactions had a final volume of 20 μL: 1 μL of primer, 10 μL of TaqMan^®^ Universal PCR Master Mix (Life Technologies, Carlsbad, CA, USA), with the last 9 μL divided between DNA extract and RNase free water according to calculated DNA concentrations to make final DNA concentration equal among samples. The reactions were performed in 96-well plates (Bio-Rad, Hercules, CA, USA), which were centrifuged for 1 min at 4000 rpm at 4 °C to remove any air bubbles. Amplification and detection were performed using an iCycler iQ™ real-time PCR detection system (Bio-Rad, Hercules, CA, USA) using the following protocol: one cycle at 95 °C for 3 min, 45 cycles at 95 °C for 30 s, at 61 °C for 30 s, and at 72 °C for 30 s. Real-time fluorescence data were collected during the annealing step. All samples as well as negative and positive controls were tested in triplicate.

To assess the possibility of false positives, PCR products were run on 1.5% agarose gels and each HSV DNA band was identified under ultraviolet transillumination.

△Ct values were obtained showing relative changes in HSV-1 shedding over time. The loading controls used in the present study were a positive control of the known concentration of viral load and negative control.

### 2.5. Statistical Analysis

The in vitro statistical analysis for cell viability assay of different concentrations of KR12 was performed using a two-way Analysis of Variance (ANOVA). Statistical significance was set at *p* ≤ 0.05. The values were measured as mean ± SD. The statistical analysis for in vitro BMDCs was performed using a two-tailed, unpaired t-test with a confidence interval of 95% for each marker (GraphPad Prism 9.2.0, GraphPad Software LLC., San Diego, CA, USA). The unit of analysis was the mouse (*n* = 6 per group).

## 3. Results

### 3.1. In Silico Comparisons of KR12 vs LL37

The structures of KR12 and LL37 are shown in Figure S1. The *rGb* of KR12 was −0.06225 while that of LL37 was −0.02632. An *rGb* ≤ 0.011 is an indication that a peptide is unstable and reactive. Both peptides were unstable and therefore reactive in aqueous solutions. However, the *rGb* of KR12 was more negative showing that it is more reactive than LL37. The MPr score of KR12 (averaged over all residues pertaining to the corresponding peptides) was approximately 1.5 times higher than that of LL37 (Figure S1). This showed that KR12 is more energetically unstable in an aqueous solution than LL37 and is therefore more reaction prone. The MPr maps of LL37 and KR12 (Figure S1) also reveal a greater extent of neutral (white) preference zones (which are unstable in water) in KR12 compared to LL37. All indicators confirm that KR12 is more highly reactive and likely has higher bioactivity than LL37. The smaller size of KR12 also allowed higher amounts of the peptide to be loaded into each nanoparticle than LL37.

### 3.2. KR12 Effects on Cells and HSV-1

**Figure 2:**
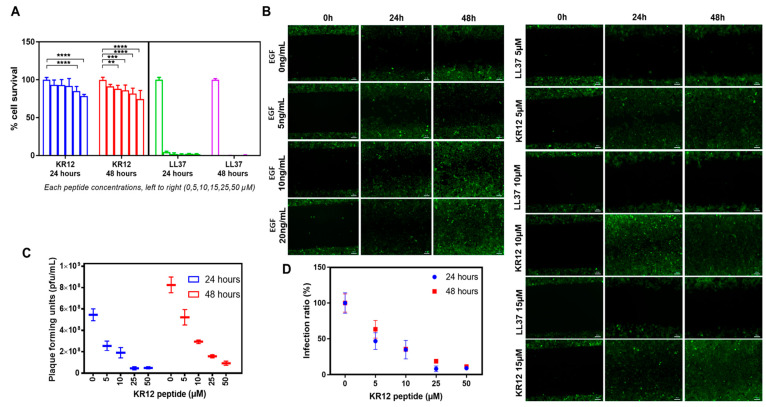
(**A**) Proliferation of HCECs with the effect of KR12 and LL37 at different concentrations. A 2-way ANOVA was used to compare the two groups at specific time point. A value of *p* ≤ 0.05 was considered statistically significant. **, *** and **** represent *p*  ≤  0.01, *p*  ≤  0.001 and *p*  ≤ 0.0001, respectively. (**B**) Cell migration and wound healing effect of different concentrations of KR12 and LL37, compared with EGF. Scale bars, 100 µm. (**C**) In vitro antiviral activity of KR 12 peptide as shown by infecting HCECs with HSV-1 (McKrae strain) at a concentration of 1.8 × 10^8^ plaque-forming units (PFU)/mL (MOI of 1) in the presence of varying concentrations of the peptide. Controls were treated with cell culture media without KR12. (**D**) Infection ratio (%) of KR 12 peptide at different concentrations was calculated as plaque numbers in treated samples/plaque numbers in untreated samples.

The first biological property measured was the rate of proliferation of HCECs in the presence of KR12 in comparison to the parent LL37 peptide. As shown in Figure 2A, all tested concentrations of LL37 displayed toxic effects at 24 and 48 h. After 24 h of exposure to KR12, only concentrations higher than 25 µM reduced the cell number significantly. However, after 48 h of exposure, KR12 concentrations higher than 10 µM showed cytotoxic effects. Nevertheless, KR12 cytotoxicity is considerably lower than that of LL37.

We also examined the ability of HCECs to undergo wound healing in the presence of KR12 and LL37. As shown in Figure 2B, the presence of KR12 did not adversely affect wound closure. KR12-exposed cells showed similar rates of wound healing as positive controls cultured in the presence of EGF. However, HCECs exposed to LL37 at all three concentrations tested (5, 10, and 15 µM) were not able to close the wound gape. These wound healing results correlate well with the cell survival and proliferation findings.

When cells were infected with HSV-1 McKrae at a multiplicity of infection (MOI) of 1 in the presence of varying amounts of KR12, the peptide markedly decreased HSV-1 activity in a dose-dependent manner as shown by the decrease in PFU compared to control infected cells without KR12 peptide at both 24 and 48 h of culture (Figure 2C). The infection ratio (%) data showed 46.42% and 64.66% infection at 5 µM, and only 34.28% and 35.55% infection at 10 µM after 24h and 48h incubation in comparison to the untreated control (Figure 2D). Infection (%) decreased gradually with increasing peptide concentration. A marked decrease was found at higher concentrations of 25 µM and 50 µM. The results showed that not all viruses are killed as more plaques formed at 48 h indicating that the remaining viruses could replicate.

#### Biocompatibility of Composite Implants with KR12

**Figure 3:**
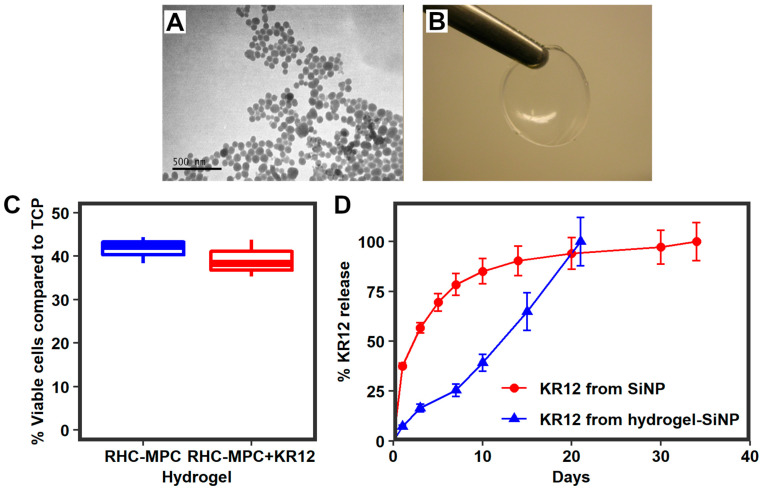
(**A**) TEM showing silica nanoparticles (SiNPs) encapsulating KR12. (**B**) RHCIII-MPC corneal implants with incorporated KR12-releasing silica nanoparticles. (**C**) MTT assay results for human corneal epithelial cell viability (HCEC), showing that the presence of KR12-SiNPs within RHCIII-MPC hydrogels did not change their capacity to support cell growth. However, cell growth on both hydrogels was less than 50% of that seen on tissue culture plastic (TCP). (**D**) Release profile of KR12 from SiNPs only and after incorporation into RHCIII-MPC hydrogels. There was no statistically significant difference between the two groups by Student’s *t*-test.

TEM showed that SiNPs containing KR12 were spherical and 50–70 nm in diameter (Figure 3A). Composite RHCIII-MPC implants with KR12-SiNPs were transparent (Figure 3B). RHCIII-MPC hydrogels with or without KR12-SiNP supported the growth of HCEC at comparable rates (Figure 3C). However, as seen in Figure 3C, at 48 h post-seeding, the number of viable HCECs on the hydrogels was less than 50% of those grown on tissue culture plastic (TCP). This was expected as MPC is an anti-fouling polymer and cells required more time for attachment and proliferation. The encapsulation efficacy of KR12 into SiNPs was 80.5%. Assuming an equal distribution of KR12-SiNPs, each implant contained 1.27 mg of KR12-SiNP. The loading efficiency was 4.8%, so each 6.75 mm diameter implant that was trephined out for grafting would therefore contain 40 µg of KR12. The composite implants released the KR12 over 3 weeks to allow uptake by cells (Figure 3D) instead of a single burst release that could have been cytotoxic and washed away by blinking.

A dendritic cell assay performed to determine immune compatibility [42] showed that the hydrogels did not activate antigen-presenting BMDCs (Figure S2). BMDCs were exposed to KR12-loaded nanoparticles, RHCIII-MPC hydrogels, or composites of both. LPS was included as a positive control of BMDCs activation. Activation was assessed by quantifying the level of expression of co-stimulatory molecules, namely CD40, CD80, and CD86, by flow cytometry. Expectedly, LPS treatment resulted in enhanced cell surface expression of all three markers on BMDCs relative to the untreated control while exposure of BMDCs to RHCIII-MPC hydrogels shows no marked changes in expression levels from the controls. Flow cytometry showed that BMDCs co-cultured with SiNPs containing KR12 died except for one sample where 2% of live cells remained. In this sample, the viable BMDCs were not activated, with a level of expression of CD40, CD80, and CD86 comparable to the unstimulated control (Figure S2). Composite RHCIII-MPC hydrogels incorporating SiNP containing KR12 induced BMDCs cytotoxicity. The cells remaining in the culture expressed low levels of CD11c, suggesting that most of them had lost a BMDCs profile or that most BDMCs did not survive the culture, precluding accurate analysis of costimulatory molecules. Although SiNP-KR12 is cytotoxic at higher doses, neither SiNP-KR12 nor RHCIII-MPC hydrogels induced the activation of BMDCs.

### 3.3. Effects of Composite K12-Releasing Implants in HSV-1-Infected Rabbit Corneas

**Figure 4:**
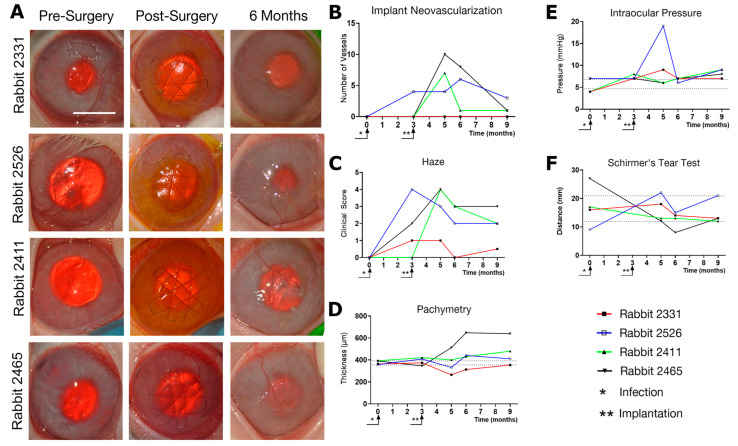
(**A**) Rabbit corneas at 3 months post-infection with HSV-1 (pre-surgery), immediately after grafting with composite RHCIII-MPC implants releasing KR12, and at 6 months post-operation. Rabbits 2411 and 2465 received an additional dose of HSV-1 during the implantation surgery to simulate virus reactivation. Representative scale bar, 5 mm. (**B**–**F**) The clinical consequences of HSV-1 infection, and subsequent post-operation outcomes after implantation of KR12-releasing corneal hydrogels. Dotted lines indicate 95% CI as calculated from preoperative data (*n* = 8) where appropriate.

In eight of the 12 rabbits, corneal infection by HSV-1 was accompanied by encephalitis at 2–6 weeks post-inoculation, and these animals were euthanized. Of the four surviving rabbits, three animals developed HSK that healed with a slight corneal haze within 7–14 days after infection (Figure 4). The fourth rabbit had moderate corneal haze, epithelial defects, and neovascularization (Figure 4).

**Figure 5:**
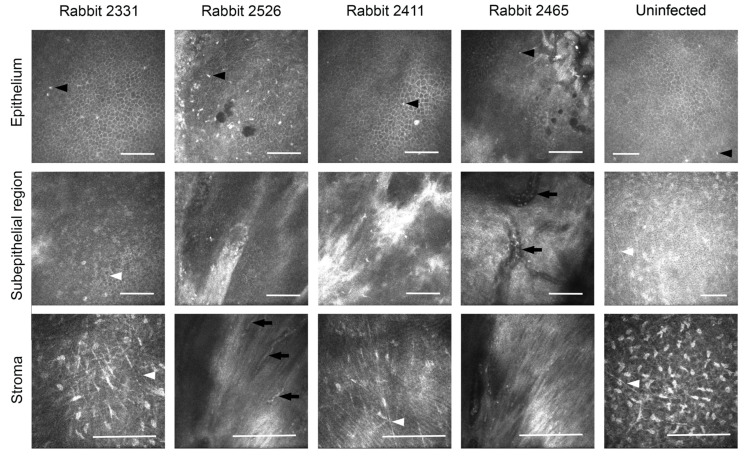
In vivo confocal microscopy images of regenerated neo-corneas after HSV-1 infection and subsequent grafting with RHCIII-MPC implants containing KR12-releasing silica dioxide nanoparticles, compared to an uninfected control cornea. The black arrowheads in epithelium indicate likely immune cells in the corneas. White arrowheads in the subepithelial and stromal images indicate nerves. Blood vessels (arrowed) were seen in the sub-epithelial region of Rabbit 2465. Scale bars, 100 µm.

**Figure 6:**
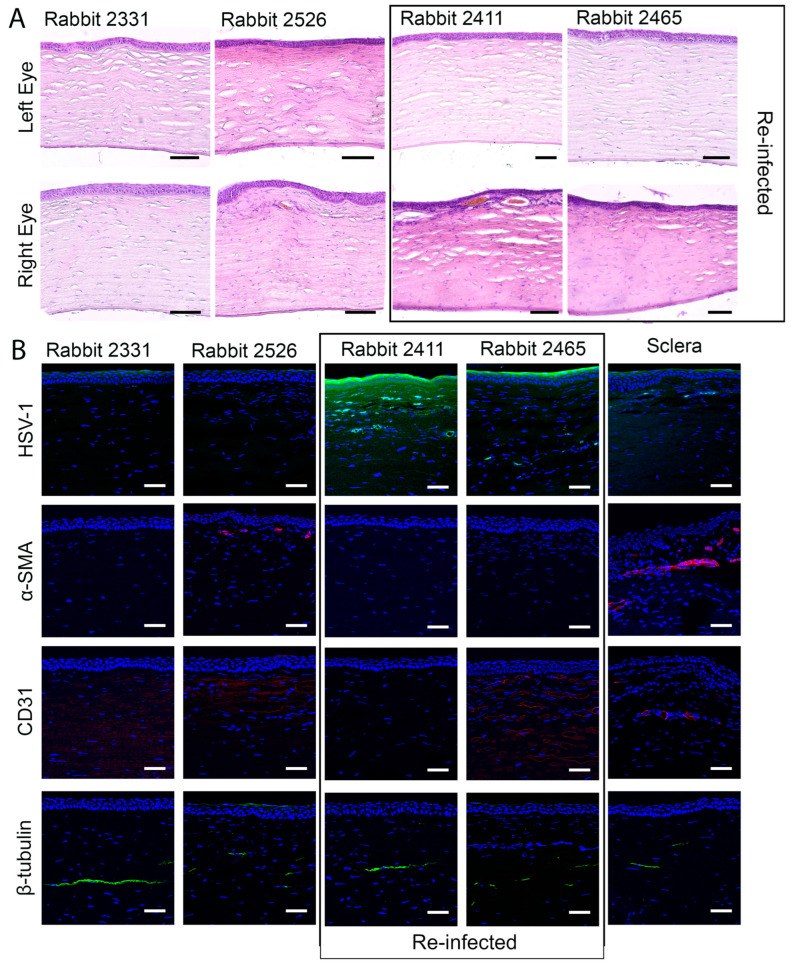
(**A**) Representative H&E sections through the regenerated neo-corneas of HSV-1-infected and implanted right eyes of rabbits, compared to the healthy, uninfected, and untreated left eyes. The unoperated eye in rabbit 2526 has increased nuclei density in the upper stroma, likely indicating viral infection. (**B**) Immunohistochemistry, showing the presence of HSV-1 virus shedding in the peri-surgically re-infected rabbits. Anti-smooth muscle actin staining showed activated fibroblasts in rabbit 2526, corresponding to the swollen appearance of the cornea in the H&E section. CD31 staining shows neovascularization in Rabbits 2526 and 2465. β-tubulin staining showed the presence of stromal nerves unaffected by the surgery but the absence of sub-epithelial nerves. Scale bars, 50 µm.

The pathologic corneas of these rabbits were excised and replaced with RHCIII-MPC implants containing KR12-releasing SiNPs by anterior lamellar surgery. To mimic viral reactivation, two rabbits were inoculated with a second virus dose of 5000 PFU immediately after surgery (rabbits 2411 and 2465). All the implants were well-tolerated, integrated seamlessly into the host corneas, and promoted stable regeneration of the corneal epithelium and stroma (Figure 4A). However, swelling and neovascularization occurred in both re-infected rabbit corneas and a third cornea (Figure 4B—Rabbits 2526, 2411, and 2465). The swelling was also seen as a haze that was confirmed by slit lamp biomicroscopy and pachymetry (Figure 4C,D). A transient increase in ocular pressure was observed in one rabbit, but by 6 months post-implantation, all intraocular pressures were within the normal range (Figure 4E). Furthermore, tear production was also within the normal range 6 months after the surgery (Figure 4F). IVCM confirmed the regeneration of the corneal epithelium and stroma (Figure 5). Small reflective cells, most likely immune cells, were seen in the epithelium. Sub-epithelial haze was seen in rabbits 2526 and 2411, while rabbit 2465 had neovascularization. The gross morphological observations were confirmed by histopathological examination of H&E sections of these corneas (Figure 6A). The untreated contralateral corneas showed uniform cell layers and no significant differences in epithelial thickness. Rabbit 2331′s treated cornea also showed normal-looking morphology.

Immunohistochemistry showed the presence of fluorescent-green-stained HSV-1 in the corneas of re-infected rabbits 2411 and 2465 (Figure 6B). The infection had also spread to the conjunctiva and the underlying sclera as shown in Figure 6B. Smooth muscle actin staining was seen around blood vessels in the positive sclera control as well as in the sub-epithelial neo-vascularized stromal area; furthermore, it showed activated fibroblasts in rabbit 2526 (Figure 6B). Furthermore, endothelial cell marker CD31 staining showed neovascularization in Rabbits 2526 and 2465 (Figure 6B).

Sub-epithelial nerves started to grow into the operated rabbit corneas at 6 months post-operation. Immunohistochemical localization of anti-β-tubulin antibody confirmed the paucity of sub-epithelial nerves, while the deeper stromal nerves that were most likely unaffected by the surgery were present (Figure 6B). PCR amplification of HSV-1 DNA extracted from tear samples collected weekly revealed that all animals shed the virus for the entire 16-week post-infection period (Figure S3) when the corneas showed symptoms of HSK. HSV-1 DNA was detected in both the infected and contralateral untreated eyes, indicating that there was a transfer of the virus between the eyes after inoculation. Virus shedding stopped at four weeks after grafting with RHCIII-MPC implants that released KR12. However, as the infected and operated corneas started shedding the virus again at 23 weeks post-operation while the untreated eye was shedding the virus at 21 weeks and again at 24 weeks, spontaneous reactivation had likely occurred (Figure S3).

## 4. Discussion

Human HSV-1 is a virus that shows reverse zoonosis and quickly infects animal models. The virus is neuroinvasive and can cause Herpes Simplex Encephalitis, which is associated with a high rate of mortality and morbidity [43]. In our study, we used the McKrae strain of HSV-1 which is commonly used to induce HSK in rabbit corneal models and is clinically relevant. However, 67% of our animals developed encephalitis despite using published viral concentrations and establishing the model in animals prior to the experiment. These animals were euthanized. HSV-1 can cause encephalitis-induced mortality in rabbits [44]. Loutsch et al. attributed the encephalitis-induced mortality in rabbits to HSV-1 reactivation after establishing latency in the trigeminal ganglia [45].

Accessibility modeling showed that the KR12 fragment of LL37 was more reactive than the parent peptide. In addition, the membrane propensity index (MPr) of KR12 further indicated that the peptide was approximately 1.5-fold more reactive than LL37 in an aqueous medium.

When tested in cell cultures, KR12 effectively blocked HSV-1 activity in a dose-dependent manner. The peptide showed cell biocompatibility in contrast to the parent LL37 peptide that was cytotoxic at all concentrations tested. HCECs cultured in the presence of KR12 showed a similar proliferative and wound healing capacity as cells cultured with 5 ng/mL of EGF. This is in keeping with previous reports that KR12 does not cause any toxicity to the cells at concentrations ranging from 32 to 512 µg/mL [46]. Our wound healing observations also showed that at low concentrations (~15 µM), KR12 stimulated comparable wound healing to EGF. The release profile of KR12 from SiNPs within RHCIII-MPC hydrogels showed a steady release over three weeks. A total of 40 µg of KR12, equivalent to 50.92 µM (molecular weight (Mw) of KR12 is 1570.95), was released over three weeks. This means that cells were exposed to lower daily doses that were conducive to wound healing, as shown in Figure 2, as opposed to a burst release if the KR12 were not encapsulated within SiNPs. Although the HCECs grown on RHCIII-MPC implants containing SiNP-KR12 showed a slower growth rate in vitro compared to those grown on tissue culture plastic, corneal re-epithelialization in the implanted rabbit corneas was not affected. Furthermore, RHCIII incorporating MPC has successfully stimulated wound healing and stable regeneration in ulcerated human corneas in a human clinical study [19]. The SiNP-based release system successfully delivered the KR12 peptide without interfering with its activity. These findings are congruent with previous data where SiO_2_ nanoparticles were used to deliver various bioactive substances [16,24].

In vitro dendritic cell assays of KR12-SiNP and RHCIII-MPC incorporating KR12-SiNP showed cytotoxicity rather than the activation of dendritic cells. These were not activated in the only samples where there were surviving BMDCs. The cytotoxicity can probably be accounted for by the burst release of KR12 in cell culture media compared to the slow release from the implants in the rabbits’ eyes over time. Alternatively, the cells could have reacted to the SiNPs, as KR12 alone had no cytotoxic effects.

There were no observable adverse immune responses in the treated rabbit corneas that could not be accounted for by the HSV-1 disease. The composite RHCIII-MPC implants incorporating KR12-SiNP had stimulated corneal epithelial and stromal tissue regeneration after the excision of the scarred tissue caused by the HSV-1 infection. Unlike previous implants of collagen-MPC in HSK mice [16], the composite implants remained stably integrated within the rabbit corneas.

Nerve regeneration began at six months post-implantation in Rabbit 2331, suggesting that KR12 slowed down or inhibited HSV-1 activity in this infected eye that showed mild disease, allowing corneal tissue and nerve regeneration within. The cornea remained clear and free from neo-vascularization. However, HSV-1 activity was only partially blocked in the other three animals as some haze and ingrowing blood vessels were observed. However, over the 6 months, there was a trend towards recovery with decreasing haze and neovascularization in all four rabbits, including the re-inoculated ones. These results are in keeping with the receding neovascularization reported in inflamed alkali-burned rabbit corneas implanted with RHCIII-MPC hydrogels [47]. In the alkali burn study, the vessels receded between six to nine months post-operation. These results suggest that the present study bears repeating in more animals, with a more extended recovery observation period to determine if the vessels seen in the operated corneas would recede over time.

PCR amplification of HSV-1 DNA showed that the KR12-releasing implants blocked viral activity, as demonstrated by the cessation of vial shedding in the collected tears. However, the virus shedding resumed at 21 weeks post-operation, suggesting that the viruses were able to reactivate. Virus reactivation showed that the released KR12 did not kill all the viruses and/or did not prevent viral latency in vivo. Furthermore, a constant level of KR12 above a threshold is likely needed for sustained antiviral activity. Reactivation is a common problem with HSV-1 corneal infections in humans and is the leading indication for the use of prophylactic drugs [10,11]. It is possible that in the future, a theranostic contact lens that can detect viral reactivation and administer an antiviral medication could help prevent reactivation [48]

KR12 possesses efficacious anti-bacterial activity [17,49,50]. We now add that the peptide also has antiviral activity against HSV-1 in vitro and in low doses, stimulated wound healing in cultured corneal epithelial cells. However, KR12’s in vivo anti-HSV-1 activity was weak and inadequate. KR12 (residues 18-29 of LL-37) is the shortest active segment of LL37. KR12 consists of only 12 amino acids containing an appropriate mixture of positive charge and hydrophobic residues to selectively inhibit viral growth. On the other hand, LL-37 is the 37-amino-acid-long peptide, containing an active fragment along with additional undesirable residues. These residues are rich in hydrophobic amino acids and reported to show non-selective lytic and cytotoxic activity towards mammalian cells [51,52,53]. Nevertheless, the composite RHCIII-MPC hydrogels allowed stable tissue and nerve regeneration even in HSV-1-infected corneas. The results show that innate CHDPs merit further study as antiviral agents for the cornea. Such natural peptides could include other derivatives of LL37 or defensins that are also produced by the human body [54], of which several, including LL37, are in clinical trials [55]. Artificial peptides developed based on CHDPs also have shown efficacious antiviral activity [56]. As natural immune system products, CHRP may avoid the viral resistance that can build up due to prolonged exposure to antiviral drugs. Furthermore, the direct delivery of antiviral peptides or other antiviral compounds into the cornea through implants might circumvent the need for high doses of antiviral medications given systemically as prophylaxis.

Limitation of the study: The innate peptide KR12 showed weak anti-viral activity in vivo. Due to the recurring nature of HSV-1 infection, viruses were found in the peri-surgical re-infected corneas after six months of the study. Nevertheless, stable regeneration and improvement were observed in all the infected corneas.

## 5. Conclusions

A significant part of the world population with corneal diseases is waiting for a donor cornea to be transplanted. Developing a biosynthetic cornea that stimulates in situ tissue regeneration offers a potential solution. In addition, many diseases and pathologies can damage corneas with varying severity. While the KR12 peptides delivered in this pilot study could not entirely block the HSV-1 activity, the composite MPC-containing implants were sufficiently robust to allow stable regeneration in the infected corneas. Our study has nonetheless demonstrated that an artificial cornea designed with delivery systems can provide a personalized solution to the patient.

## Figures and Tables

**Table 1:** Antibodies for flow cytometry.

Target	Antibody	Dilution Factor
CD11c	Brilliant Violet 650™ anti-mouse CD11c, Clone: N418, IsoType: Armenian Hamster IgG, Format: BV650, APP: FC, BioLegend (San Diego, CA, USA), 117339	1:600
CD40	CD40, APC, clone: 1C10, eBioscience™ (Carlsbad, CA, USA), 501129392	1:400
CD80	PE anti-mouse CD80, Clone: 16-10A1, IsoType: Armenian Hamster IgG, Biolegend (San Diego, CA, USA), 104708	1:1600
CD86	FITC anti-mouse CD86, Clone: GL-1, IsoType: Rat IgG2a,κ, Biolegend (San Diego, CA, USA), 105006	1:50

**Table 2:** Antibodies for immunohistochemistry.

Target	Antibody	Dilution Factor
herpes simplex virus serotype 1	Anti-HSV1 [20.7.1], ab860 Abcam, Cambridge, UK	1:100
activated stromal cells or myofibroblast	Anti-SMA, [1A4] ab7817, Abcam, Cambridge, UK	1:50
vascular endothelium	Anti-CD31, ab199012, Abcam, Cambridge, UK	1:50
lymphatic vessels	Anti-Lyve 1, ab14917, Abcam, Cambridge, UK	1:100
Macrophages	Anti-MAC 387, ab22506, Abcam, Cambridge, UK	1:100
Nerves	neuron-specific Anti-beta III Tubulin [Tu-20], ab7751, Abcam, Cambridge, UK	1:100

## Data Availability

The raw data supporting the conclusions of this article will be made available by the authors upon request.

## Supplementary Materials

The following supporting information can be downloaded at: https://www.mdpi.com/article/10.3390/pharmaceutics15061658/s1, Figure S1: In silico structure-driven stability estimates for LL37 and KR12: Figure S2: Histogram of CD40, CD80, and CD86 expression in BMDCs under various conditions; Figure S3: Quantitative PCR performed on tears collected from rabbits infected with HSV-1and subsequently grafted over 24 weeks (6 months post-operation).

## Abbreviations

uM	micro Molar
α-SMA	Alpha smooth muscle actin
β-tubulin	Beta-tubulin
ASA	Accessible surface areas
ACV	Acyclovir
APC	Antigen-presenting cells
APS	Ammonium persulphate
BMDCs	Bone marrow dendritic cells
Bur	Burial
CHDPs	Cationic host defense peptides
CI	Confidence Interval
CD	Cluster of differentiation
DNA	Deoxyribonucleic acid
DMEM	Dulbecco’s Modified Eagle Medium
*E. coli*	*Escherichia coli*
EDC	N-(3-dimethylaminopropyl)-N’-ethylcarbodiimide
EGF	Epidermal growth factor
FBS	Fetal bovine serum
FITC	Fluorescein isothiocyanate
GM-CSF	Granulocyte-macrophage colony-stimulating factor
GFP	Green fluorescence protein
GFP	HCECs Green fluorescence protein transfected human corneal epithelial cells
HEPES	(4-(2-hydroxyethyl)-1-piperazineethanesulfonic acid)
H&E	Hematoxylin-eosin
HCECs	Human corneal epithelial cells
HSV-1	Herpes Simplex Virus serotype 1
Ig G	Immunoglobulin G
IOP	Intraocular pressure
IVCM	In vivo confocal microscopy
KR12	KRIVQRIKDFLR
KSFM	Keratinocyte serum free media
LPS	Lipopolysaccharides
MTT	3-(4,5-Dimethylthiazol-2-yl)-2,5-Diphenyl Tetrazolium Bromide
MPr	Membrane propensity index
Mw	Molecular weight
MES	Morpholinoethane sulfonic acid monohydrate
MOI	Multiplicity of infection
NHS	N-hydroxysuccinimide
PFA	Paraformaldehyde
PFUs	Plaque-forming units
PCR	Polymerase chain reaction
PBS	Phosphate-buffered saline
PEGDA	Poly (ethylene glycol) diacrylate
RHCIII-MPC	Recombinant human collagen type III and 2-methacryloyloxyethyl phosphorylcholine
*rGb*	Residue Given burial
RT	Room temperature
SiNPs	Silica dioxide nanoparticles
TEM	Transmission electron microscopy
TEMED	N,N,N,N-tetramethylethylenediamine
TCP	Tissue culture plastic (TCP)
