# Supplementary material for: Phosphorylcholine and KR12-Containing Corneal Implants in HSV-1-Infected Rabbit Corneas
Source: Pharmaceutics. 2023 Jun 5;15(6):1658. doi: 10.3390/pharmaceutics15061658 (PMC10305192; doi:10.3390/pharmaceutics15061658)
Supplement: Supplementary file 1 [file pharmaceutics-15-01658-s001.zip › pharmaceutics-2270209-supplementary.pdf]

Supplementary Material

Phosphorylcholine and KR12-containing Corneal Implants in HSV-1 infected Rabbit Corneas

Kamal Malhotra[1,2,†], Oleksiy Buznyk[3,4,†], Mohammad Mirazul Islam[3,§], Elle Edin[1,2,3,5], Sankar Basu[6], Marc Groleau[1,2,6], Delali Shana Dégué[1,2,5], Per Fagerholm[3], Adrien Fois[2,7], Sylvie Lesage[2,7], Jaganmohan R. Jangamreddy[3,ǂ], Egidijus Šimoliūnas[8], Aneta Liszka[3], LiQD Cornea Consortium[9], Hirak K. Patra[3,10,*], May Griffith[1,2,3,5,]

[1]Department of Ophthalmology, Université de Montréal, Montreal, Quebec, Canada.

[2]Maisonneuve-Rosemont Hospital Research Centre, Montreal, Quebec, Canada

[3]Department of Clinical and Experimental Medicine, Linköping University, Linköping, Sweden

[4]Filatov Institute of Eye Diseases and Tissue Therapy of the NAMS of Ukraine, Odessa, Ukraine

[5]Institute of Biomedical Engineering, Université de Montréal, Montreal, Quebec, Canada

[6]Department of Microbiology, Asutosh College, Affiliated with University of Calcutta, Kolkata, India

[7]Département de Microbiologie, Infectiologie et Immunologie, Université de Montréal, Montreal, Quebec, Canada

[8]Department of Biological Models, Institute of Biochemistry, Life Sciences Center, Vilnius University, Vilnius, Lithuania

[9]Euronanomedicine 3 LiQD Cornea Consortium (May Griffith, Sylvie Lesage, Maisonneuve-Rosemont Hospital Research Centre, Quebec, Canada; Illimar Altosaar, Evelin Loit, Estonian Univ. of Life Sciences, Estonia; Olivier Zelphati, OZ Biosciences, France; Virginija Bukelskienė, Rimvydas Ašoklis, Vilnius University, Lithuania)

[10]Department of Surgical Biotechnology, UCL Division of Surgery and Interventional Science, University College London, London, UK

Supplementary Figures

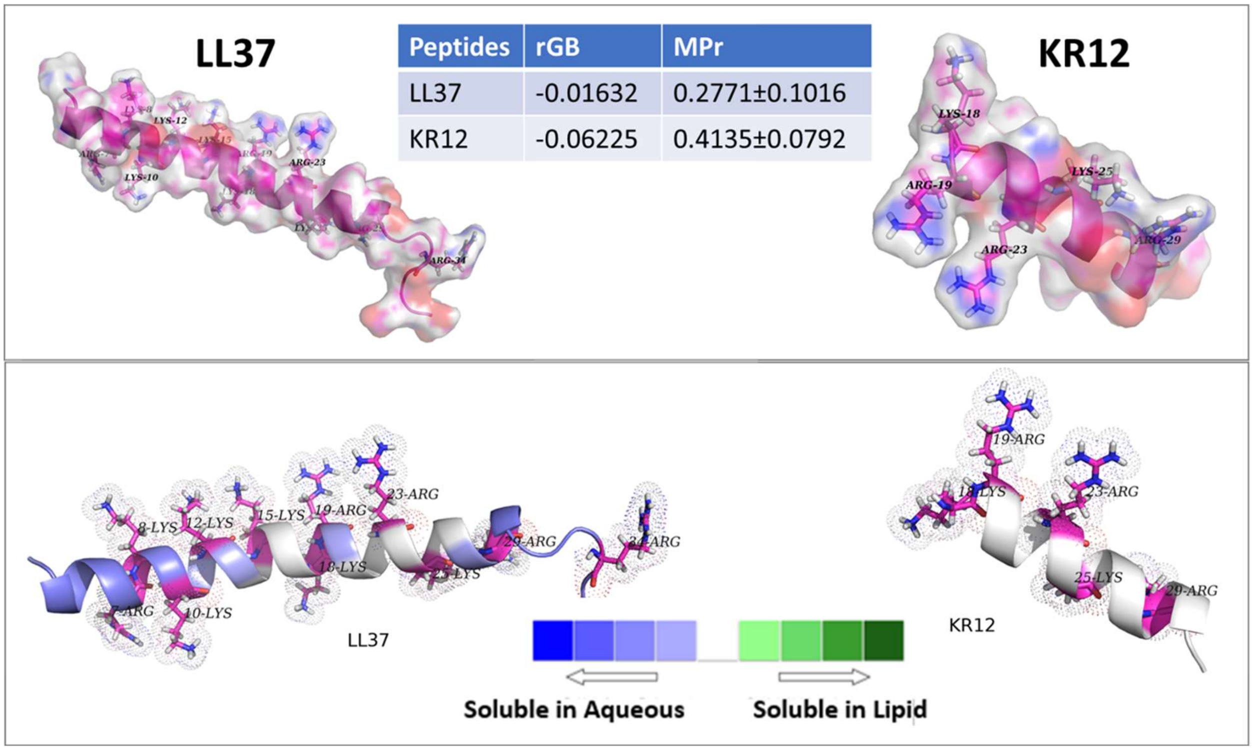

Fig. S1. ***In silico*** **structure-driven stability estimates for LL37 and KR12.** The top panel portrays the solvent-accessible surfaces (colored based on atom types) for LL37 (left) and KR12 (right) corresponding to their accessibility (rGb) scores. The bottom panel shows the colour-mapped structures derived from BRANEart in the same order for the two peptides, colored based on their membrane propensity (MPr) indices (as illustrated in the colour bars). For all the panels, positively charged residues (Lys+, Arg+) are highlighted as sticks on cartoon presentations off the mainchain helices. For the bottom panel (MPr), the positively charged residues are further encapsulated by their van der Waals surfaces (drawn in dots) to portray their strategic positions on these structures.

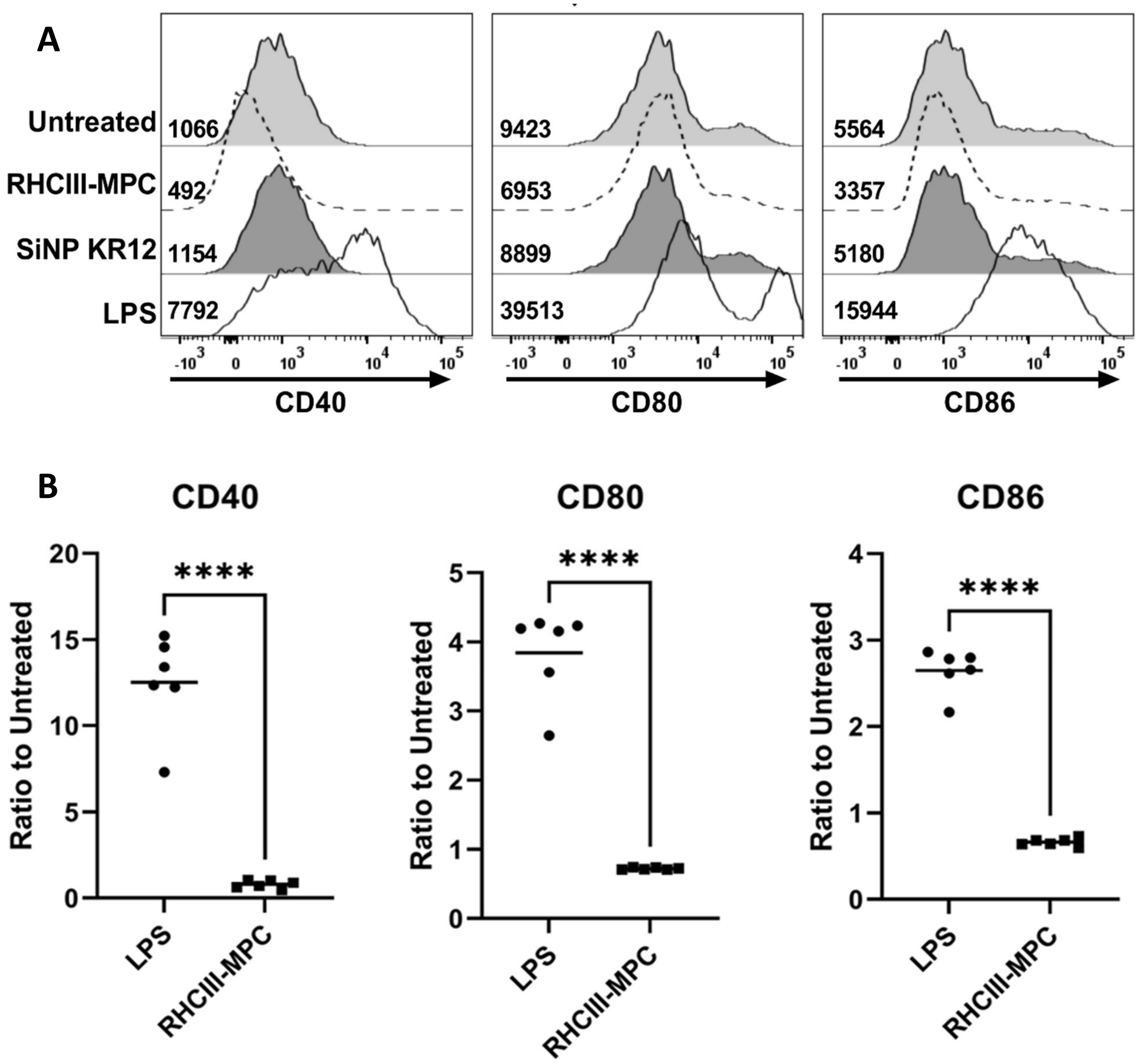

Fig. S2. Histogram of CD40, CD80 and CD86 expression in BMDCs under various conditions. A) Untreated samples and those exposed to RCHIII-MPC, silica nanoparticles (SiNPs) releasing KR12 (SiNP KR12) and LPS, as indicated. The numbers on the histogram indicate the mean fluorescence intensity. The data are shown for the only culture where BMDCs survived the SiNP KR12 treatment. B) Expression of pro-inflammatory molecules in BMDCs after exposure to RHCIII-MPC hydrogels. Flow cytometry was used to measure the expression of CD40, CD80 and CD86 in BMDCs. Data is presented as a ratio of mean fluorescence intensity of the treated cells compared to the untreated BMDCs. LPS was used as a positive control for BMDC activation. Live cells that expressed CD11c were used for the dendritic cell counts using a flow cytometer. ****P < 0.0001 by Student’s t test.

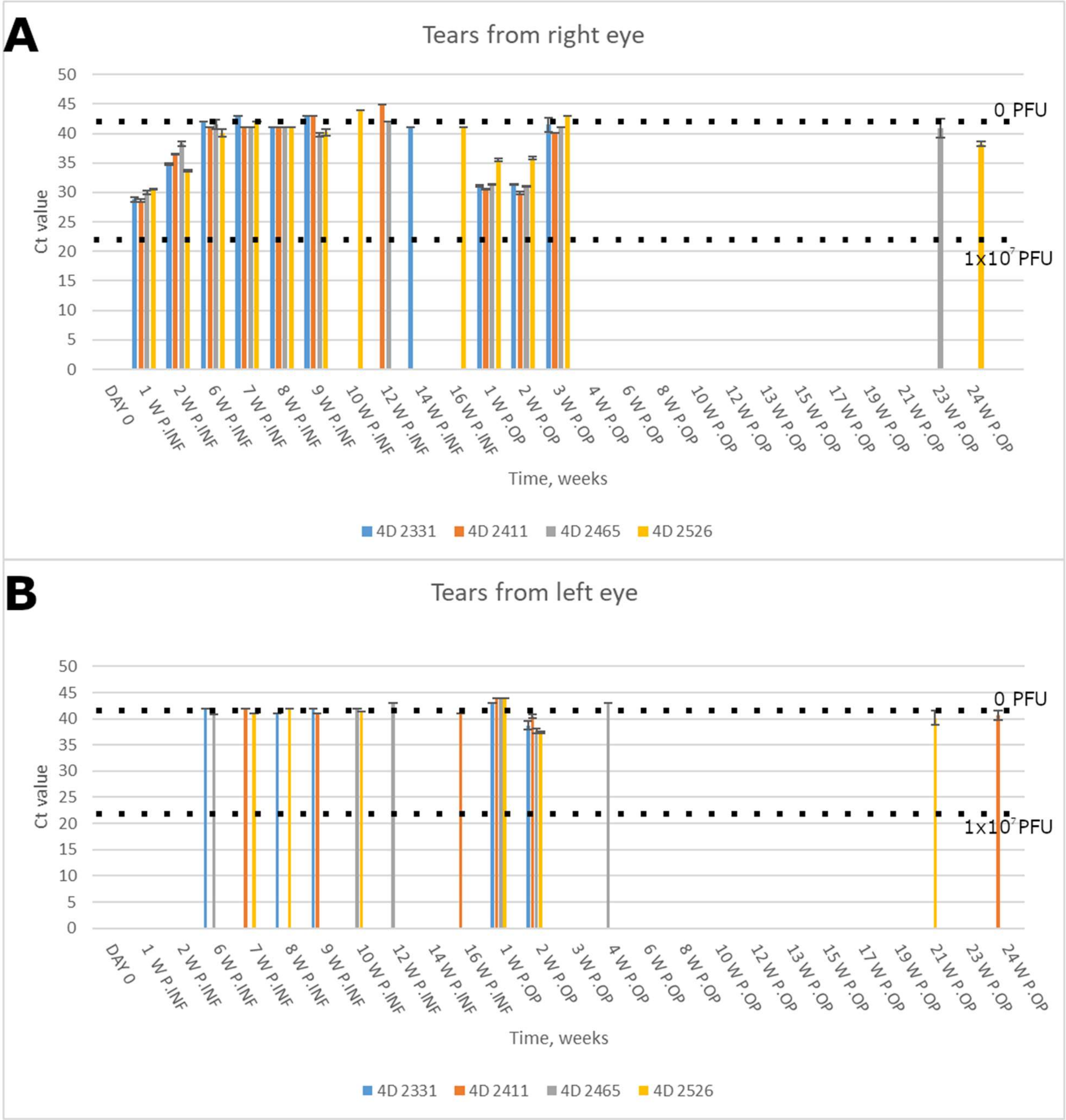

Fig. S3. Quantitative PCR performed on tears collected from rabbits infected with HSV-1 and subsequently grafted over 24 weeks (6 months post-operation). A - HSV-1 viral load in the tears from the right (operated) and B – from the left eye. The detectable amounts of HSV-1 ICP 27 product are given for both the infected and subsequently grafted right corneas compared to the untreated left corneas. The spread of the virus to the contralateral eyes is seen at 6 weeks post-infection. Virus shedding tapers off by week 4 post-operation but spontaneous virus reactivation from 21-24 weeks post-operation was observed in three animals in both right and left eyes. The

dotted lines show Ct values from positive ($1x10^7$ plaque forming units (PFU)), and negative (0 PFU) controls.
